# Supplementary material for: Identifying repeat domains in large genomes
Source: Genome Biol. 2006 Jan 31;7(1):R7. doi: 10.1186/gb-2006-7-1-r7 (PMC1431705; doi:10.1186/gb-2006-7-1-r7)
Supplement: Additional File 1 — A zipped file of browsable HTML files with a complete list of the connected components in the repeat domain graph of human Repbase. [file gb-2006-7-1-r7-S1.gz › html/subgraphs/8845.html]

|  |  |
| --- | --- |
| id | repbase name |
| 120 | MER31 |
| 133 | MER67A |
| 134 | MER67B |
| 135 | MER67C |
| 186 | LTR23 |
| 195 | MER31A |
| 196 | MER31B |
| 197 | MER67D |
| 340 | LTR56 |
| 521 | LTR70 |
